# Daily Activity Measured With Wearable Technology as a Novel Measurement of Treatment Effect in Patients With Coronary Microvascular Dysfunction: Substudy of a Randomized Controlled Crossover Trial

**DOI:** 10.2196/resprot.8057

**Published:** 2017-12-20

**Authors:** Kade Birkeland, Raj M Khandwalla, Ilan Kedan, Chrisandra L Shufelt, Puja K Mehta, Margo B Minissian, Janet Wei, Eileen M Handberg, Louise EJ Thomson, Daniel S Berman, John W Petersen, R David Anderson, Galen Cook-Wiens, Carl J Pepine, C Noel Bairey Merz

**Affiliations:** ^1^ Cedars-Sinai Medical Care Foundation Cedars-Sinai Medical Center Los Angeles, CA United States; ^2^ Cedars-Sinai Medical Group Cedars-Sinai Heart Institute Los Angeles, CA United States; ^3^ Barbra Streisand Women’s Heart Center Cedars-Sinai Heart Institute Los Angeles, CA United States; ^4^ Division of Cardiology Emory University Atlanta, GA United States; ^5^ Division of Cardiology University of Florida Gainesville, FL United States; ^6^ S Mark Taper Foundation Imaging Center Cedars-Sinai Medical Center Los Angeles, CA United States; ^7^ Biostatistics and Bioinformatics Research Center Cedars-Sinai Medical Center Los Angeles, CA United States

**Keywords:** angina, coronary microvascular dysfunction, physical activity

## Abstract

**Background:**

Digital wearable devices provide a “real-world” assessment of physical activity and quantify intervention-related changes in clinical trials. However, the value of digital wearable device-recorded physical activity as a clinical trial outcome is unknown.

**Objective:**

Because late sodium channel inhibition (ranolazine) improves stress laboratory exercise duration among angina patients, we proposed that this benefit could be quantified and translated during daily life by measuring digital wearable device-determined step count in a clinical trial.

**Methods:**

We conducted a substudy in a randomized, double-blinded, placebo-controlled, crossover trial of participants with angina and coronary microvascular dysfunction (CMD) with no obstructive coronary artery disease to evaluate the value of digital wearable device monitoring. Ranolazine or placebo were administered (500-1000 mg twice a day) for 2 weeks with a subsequent 2-week washout followed by crossover to ranolazine or placebo (500-1000 mg twice a day) for an additional 2 weeks. The outcome of interest was within-subject difference in Fitbit Flex daily step count during week 2 of ranolazine versus placebo during each treatment period. Secondary outcomes included within-subject differences in angina, quality of life, myocardial perfusion reserve, and diastolic function.

**Results:**

A total of 43 participants were enrolled in the substudy and 30 successfully completed the substudy for analysis. Overall, late sodium channel inhibition reduced within-subject daily step count versus placebo (mean 5757 [SD 3076] vs mean 6593 [SD 339], *P*=.01) but did not improve angina (Seattle Angina Questionnaire-7 [SAQ-7]) (*P*=.83). Among the subgroup with improved angina (SAQ-7), a direct correlation with increased step count (r=.42, *P*=.02) was observed.

**Conclusions:**

We report one of the first studies to use digital wearable device-determined step count as an outcome variable in a placebo-controlled crossover trial of late sodium channel inhibition in participants with CMD. Our substudy demonstrates that late sodium channel inhibition was associated with a decreased step count overall, although the subgroup with angina improvement had a step count increase. Our findings suggest digital wearable device technology may provide new insights in clinical trial research.

**Trial Registration:**

Clinicaltrials.gov NCT01342029; https://clinicaltrials.gov/ct2/show/NCT01342029 (Archived by WebCite at http://www.webcitation.org/6uyd6B2PO)

## Introduction

Mobile wireless devices and connected wearable biosensors have the potential to provide new insights into chronic medical conditions and help clinicians develop personalized treatment strategies. One potential application of digital wearable device technology is the real-time evaluation of change in daily activity as a clinical outcome. Patient activity level is traditionally assessed using surrogate measures such as exercise testing or patient questionnaires. However, these tools are limited to point-in-time observation and questionnaires provide subjective information that is retrospective. Furthermore, although patient questionnaires correlate with exercise stress testing [1], they may fail to correlate with wearable pedometer-measured daily activity [2], which along with research-grade accelerometer-measured daily activity, have generated physical activity outcome variables for clinical trials in the general population as well as patients at risk for coronary artery disease (CAD) or diagnosed with CAD [3-6]. Commercially available, digital wearable devices are capable of accurately [7-11] measuring daily activity (step count) continuously and objectively, including in patients with cardiac disease [10]. Compared to pedometers and research-grade accelerometers [7-11], digital wearable devices are novel because they generate daily activity data in real time via synchronization to a connected computer or mobile device, which allows for new insights into treatment response and, in particular, how treatments may impact patients in their daily lives.

One area for such potential is the management of angina pectoris, a chronic medical condition with symptoms that decrease exercise capacity, decrease quality of life (QoL), and lead patients to limit physical activities in daily life [12-15]. In treating angina, therapeutic goals include optimizing level of physical activity, functional capacity, and QoL by eliminating ischemic symptoms [16]. Fortunately, treatment of angina with antianginal pharmacologic therapy, cardiac rehabilitation, or psychological intervention has been shown to improve QoL and increase level of physical activity [17-19]. However, until recently, evaluation of the response to antianginal therapies among patients with obstructive CAD has focused on laboratory exercise testing, nitroglycerin utilization, or retrospective QoL and daily activity data capture using patient diaries and questionnaires. It is now recognized that a significant portion of patients with angina are women without obstructive CAD. Many of these women have coronary microvascular dysfunction (CMD) that is their major mechanism for myocardial ischemia. It is estimated to affect at least 2 to 3 million Americans, most frequently women, and has been found to not only decrease QoL and lead to limitation of physical activities, but also increase the risk of death and other cardiovascular events, including heart failure (diastolic dysfunction), myocardial infarction, and stroke [12-15,20-22]. Although specific pathophysiological mechanism(s) of CMD are not fully understood, when evidence of ischemia is present, CMD is treated with conventional antiischemic, antianginal pharmacologic therapies such as beta blockers, calcium channel blockers, and nitrates with the intention being to relieve anginal symptoms so that QoL improves, including allowing for increased exercise capacity, QoL, and physical activity [20]. Unfortunately, these traditional therapies were developed and approved over past decades largely in patients with obstructive CAD and “reproducible” exercise test electrocardiogram (ECG) results. Patients without obstructive CAD often do not have these “reproducible” exercise test results and also do not reliably respond to these traditional antianginals. So these patients continue to have angina, which decreases their ability to be physically active in their daily lives and, as such, they become sedentary as they continue to suffer from symptoms that diminish their QoL and daily activity with no readily available therapy or novel methods to evaluate their responses to treatment, representing an important knowledge gap [12-15,20].

Late sodium channel inhibition (ranolazine) is an antiischemic pharmacologic therapy indicated for the treatment of angina due to CAD that has been demonstrated to improve physical activity, exercise duration, and QoL [17,23,24]. However, there is very limited knowledge about its efficacy and safety in the treatment of CMD [23-27]. A pilot study with ranolazine versus placebo in 20 women with CMD suggested that ranolazine improved angina [28]. Similarly, results of two small studies [17,29] (58 and 46 participants with no obstructive CAD) also supported ranolazine efficacy for increasing coronary flow reserve (CFR) with one study demonstrating improved QoL and physical activity [17]. However, in a much larger (N=128) randomized, placebo-controlled crossover trial of patients with CMD, which aimed to mechanistically test if angina symptoms were related to cardiac magnetic resonance imaging (MRI) myocardial perfusion reserve index (MPRI), late sodium current inhibition with ranolazine did not improve angina, QoL, ischemia, or diastolic function [30]. These discordant findings were likely related to small sample sizes, differences in participant characteristics (typical male “effort” angina vs female nontypical angina), and evaluation methodologies (exercise vs pharmacological testing, directly vs indirectly measured CFR), as well as severity of CMD [30].

Angina clinical trials have used exercise laboratory testing, 6-minute walk, ECG monitoring, cardiac imaging, QoL questionnaires, and pedometers to determine if treatment of ischemia with antianginal therapy relieves symptoms and results in improved physical activity, functionality, and QoL. The totality of evidence suggests that treatment of angina can relieve symptoms and that no one therapy is more effective than the other. However, beyond larger modern studies that have used questionnaires or historical small studies that used pedometers, our understanding of how treatment with antianginal therapy affects daily activity is limited. As such, data generated by digital wearable devices has the potential to provide new insights into the efficacy of antianginal therapies as it relates to physical activity in daily life in patients with angina, particularly in the CMD population.

To further explore use of digital technology in a therapeutic cardiovascular trial, we conducted a substudy within a randomized, double-blinded, placebo-controlled, crossover trial of patients with angina and CMD and no obstructive CAD [30] to assess within-subject change in daily activity measured by mean daily step count using the Fitbit Flex wireless activity monitor. Specifically, the objective of this study was to determine if treatment of angina with ranolazine in patients with CMD would provide symptomatic relief sufficient to allow patients to increase their levels of daily activity. We hypothesized that those treated with ranolazine compared to placebo would have a higher mean daily step count, and that those with greater antianginal responses would have greater step count increases.

## Methods

### Patient Population

Participants were recruited from the two-site parent trial [30], which was conducted at both Cedars-Sinai Medical Center and the University of Florida, Gainesville, FL. Patients enrolled in the parent trial had symptoms and signs of myocardial ischemia, no obstructive CAD, and CMD as measured by invasive CFR or noninvasive cardiac MRI-determined MPRI. Inclusion and exclusion criteria were the same as the parent trial [30], with the additional exclusion criteria of immobility and/or physical inability to wear the wristband. Patient characteristics and demographics were collected at baseline and included classification of patients with typical angina or nontypical angina on screening and enrollment. Typical angina was defined as substernal chest pain precipitated by physical exertion or emotional stress and relieved with rest or nitroglycerin; nontypical angina was defined as symptoms that did not meet criteria for typical angina. In addition, participants who underwent invasive CFR were classified as CFR either less than 2.5 or 2.5 and greater. Institutional Review Boards at Cedars-Sinai Medical Center and the University of Florida approved the study, and all participants gave written informed consent before participation.

### Study Design

**Figure 1:**
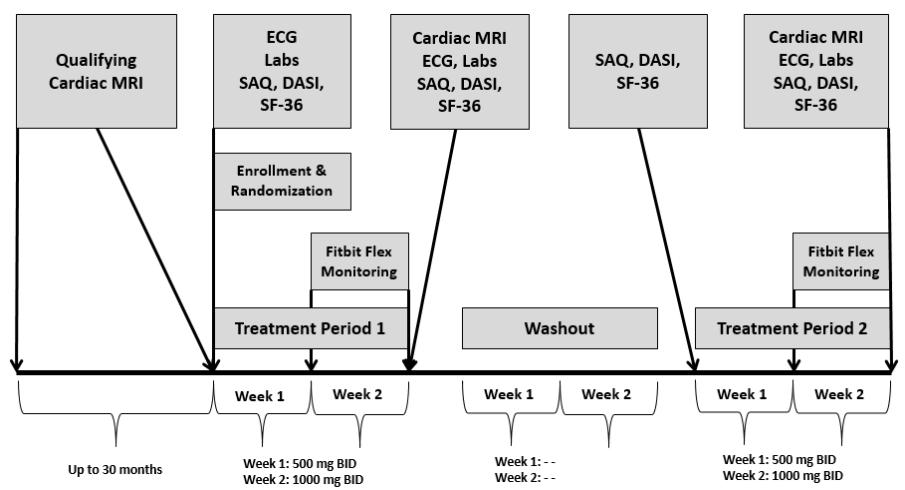
Substudy design flow diagram, participant screening, enrollment, and randomization flow diagram. Treatment period 1 and 2 with daily activity monitoring during week 2 of each treatment period: randomized to sequence of ranolazine first followed by crossover to placebo or vice versa. BID: twice a day; DASI: Duke Activity Status Index; ECG: electrocardiogram; MRI: magnetic resonance imaging; SAQ: Seattle Angina Questionnaire; SF-36: 36-item Short Form Survey.

The parent trial was a double-blind, placebo-controlled, crossover trial with short-term (2 week treatment periods; week 1: ranolazine or placebo 500 mg twice a day, week 2: ranolazine or placebo 1000 mg twice a day) ranolazine-placebo exposure (order randomly assigned to a sequence of ranolazine or placebo first followed by a 2-week washout and subsequent crossover to placebo or ranolazine or vice versa) [30]. The substudy design included daily activity monitoring with a digital wearable device performed during week 2 of both treatment period 1 and 2 (ranolazine 1000 mg twice a day or placebo 1000 mg twice a day) (Figure 1) [30].

The duration of daily activity monitoring was designed for 1-week duration to account for daily activity that occurs in real life. In addition, it was previously demonstrated that ranolazine increases exercise performance in patients with angina-limited exercise after 1 week of treatment [23]. Daily activity monitoring occurred during week 2 of each 2-week treatment period instead of during week 1 because low-dose study treatment (500 mg twice a day) was initiated during week 1 and titrated to high-dose study treatment (1000 mg twice a day) during week 2, such that daily activity monitoring during week 2 of each treatment period would assess daily activity under the maximal therapeutic effect of ranolazine or placebo. Daily activity was quantified as step counts and categorized by pedometer-determined indexes of physical activity in healthy adults as follows: (1) sedentary lifestyle index: less than 5000 steps per day; (2) low active: 5000 to 7499 steps per day; (3) somewhat active: 7500 to 9999 steps per day; (4) active: 10,000 or more steps per day; and (5) highly active: greater than 12,500 steps per day [31].

### Digital Wearable Device

The wearable accelerometer was the Fitbit Flex, which continuously measures daily physical activity that is reported as step count [8]. The Fitbit Flex has a battery life of 5 days and generates data that includes steps, distance covered (miles), and calories burned [8]. At the time of study design, prior versions of the Fitbit device had been reported to have an accuracy of 95% to 97% for measuring activity (steps) when worn correctly, and accuracy was evaluated by direct observation and by comparison to other activity monitors [7,9]. The Fitbit Flex has since been validated for measuring physical activity, including in patients with cardiac disease [10,11]. The Fitbit Flex was selected so that the wearable accelerometer would be encased in a wristband with the assumption being that this would decrease the risk for loss of device.

Before randomization, each participant was provided with a Fitbit Flex, a dongle for wireless synchronization, a USB cord, and a wall charger. Participants were instructed regarding the device placement and the daily physical activity monitoring protocol, which included scheduled monitoring dates, device charging, wrist placement, and device synchronization. Participants were encouraged not to modify their daily activity or achieve any specific step count goal or daily activity level. The device was worn by the participant on the nondominant wrist continuously each day during week 2 of both the active treatment and placebo periods. All days with step count data were considered a valid day of wear as long as the participant did not report any violations of the protocol in that they continuously wore the Fitbit per protocol during the entirety of the monitoring period. Research coordinators verbally confirmed that participants complied with the protocol after completion of each monitoring period. Per protocol, the device could only be removed temporarily for bathing or swimming, but otherwise removal was not allowed unless a participant was instructed to remove the device by the research coordinator (ie, adverse device reaction, device failure, or device charging while sleeping if indicated).

Digital wearable device data were uploaded regularly by participants via computer synchronization using the “Sync Now” function in the Fitbit Connect software, which was installed on each participant’s personal computer or device and aggregated by research coordinators for analysis. Each device was accompanied by a unique account that was password protected and accessible only to research coordinators; therefore, participants were not able to view their own step count data. They were able to view the Fitbit Flex indicator lights, although they were not instructed as to what the specific meaning of the light patterns indicated. During the clinical trial, participants received reminder telephone calls regarding device charging, placement, and synchronization. In the case that a research coordinator determined that a device needed to be charged, the participant was instructed to remove the device during sleep and place the device on the nondominant wrist immediately on waking. Research coordinators monitored device synchronization and intervened if the device failed to upload data during monitoring periods. To assure participant safety and equipment integrity, participants were also instructed about allergic reactions, exposure to liquids, and device cleaning. In total, 15 Fitbit Flex’s purchased between 2013 and 2015 were used throughout the duration of the study.

### Angina and Quality of Life Questionnaires

Patient questionnaires were administered as described [30] to assess angina (Seattle Angina Questionnaire [SAQ] and SAQ-7), functional capacity/status, and QoL (Duke Activity Status Index [DASI] and the 36-item Short Form Survey [SF-36]). Angina was also assessed by an angina and nitroglycerin use diary as previously described in the parent trial [30].

### Cardiac Magnetic Resonance Imaging

The cardiac MRI protocol was performed as previously described and was conducted under identical conditions and timing, dosing, and settings, approximately 4 hours after the morning study drug dose [30,32-35]. A cardiac MRI MPRI of 1.8 or less was considered abnormal [33] and correlates with invasive coronary reactivity testing [34] and risk factors [35]. MPRI data were obtained to evaluate CMD, and left ventricular peak filling rate (PFR) and time to PFR (tPFR) data were obtained to evaluate diastolic function [36].

### Invasive Coronary Reactivity Testing

Clinically indicated invasive coronary reactivity testing [21,30,37] measured coronary microvascular and macrovascular endothelial and non-endothelial-dependent function was available for 13 of 30 participants (43%) as previously described [38].

### Statistical Analysis

This substudy was planned to enroll 30 participants to achieve 80% power to detect a mean difference in daily step count between a patient’s ranolazine and placebo treatments of 1000 using a paired *t* test with the crossover design of the parent study, at a significance level of .05, assuming a standard deviation of 1863 in step count difference. Participants were randomized to two sequences (ranolazine then placebo or placebo then ranolazine) centrally at a 1:1 ratio and blocked by clinical site [39].

The analytic approach was a within-subject comparison (paired) of the difference between a participant’s mean daily step count during 1 week on ranolazine and their mean daily step count during 1 week on placebo. The main endpoint of the parent trial was the difference between change in SAQ for ranolazine versus placebo. There were two treatment periods, ranolazine and placebo, giving two measurements per participant for mean step count and cardiac MRI variables. There were a total of four measurements per participant, including treatment baselines and posttreatment values, for SAQ and QoL giving two treatment changes per participants. The primary approach was a standard paired *t* test. Difference in mean daily step count was also evaluated for the subgroups of typical or nontypical angina, CFR of less than 2.5, and “low active” or “sedentary” daily physical activity during placebo. Linear regression models like those used in the parent study [38] were tested using within-subject treatment differences as the outcome (treatment change in step count) to adjust for potential interaction of relevant clinical variables with treatment (examine how other variables influenced the effect the drug had on step count), where data were available, and to explore subgroup effects for angina type (typical angina or nontypical angina). Carryover effects tested the interaction between treatment and period by comparing the within-subject mean with means between the arms [40]. The significance level was set to .05. A participant was included if they had at least 3 days of 7 days of step count data available in each treatment period. If data were missing such that a participant did not have a minimum of 3 of 7 days of step count data, they were not included per protocol in the analysis. A minimum of 3 days of step count data was required in each treatment period based on pedometer daily activity monitoring findings reported by Tudor-Locke et al [41], who determined that 3 days of step count data provide a sufficient estimate of steps per day during a 7-day monitoring period. Analyses were performed using SAS version 9.3 (SAS Institute, Inc, Cary, NC, USA).

### Study Oversight

The study was an investigator-initiated, intramurally funded substudy embedded within a parent trial ancillary to the National Heart, Lung, and Blood Institute (NHLBI)-sponsored Women’s Ischemia Syndrome Evaluation (WISE) study. The parent trial was funded, in part, by Gilead Sciences and was overseen by the WISE Data Safety Monitoring Committee. Statistical analysis was performed by investigators independent of NHLBI and Gilead, masked to treatment assignment. The decision to submit for publication was made by investigators who had access to all data after the last participant completed the study.

## Results

### Participant Characteristics

**Figure 2:**
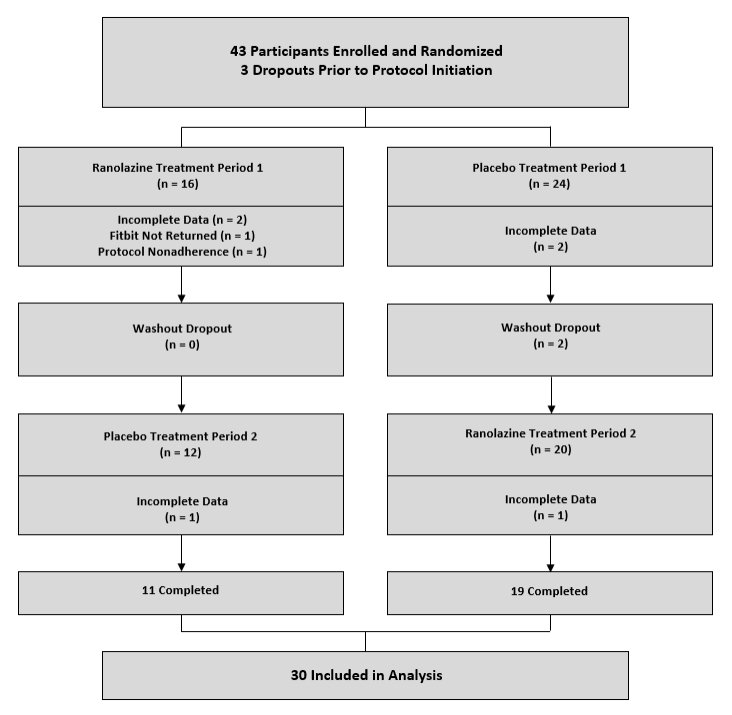
Substudy participant screening, enrollment, randomization, and completion flow diagram. Participants were randomized to sequence of ranolazine first followed by crossover to placebo (treatment period 1) or vice versa (treatment period 2). For both treatment periods 1 and 2, daily activity monitoring occurred during week 2.

Between February 12, 2014 and June 1, 2016, 43 participants entered the Fitbit pilot study, 31 of whom were also enrolled in the parent trial (29 included in the parent trial analyses), and 12 who were enrolled in the substudy after the parent trial closed enrollment. Among the 43 participants who entered the substudy, 13 participants (30%) could not be analyzed for within-subject change in step count because of dropout before protocol initiation (n=3), incomplete data (n=6), Fitbit Flex not returned (n=1), protocol nonadherence (n=1), and washout dropout (n=2), leaving 30 participants (70%) who adhered to the protocol sufficient to provide adequate data for analysis as an outcome variable (Figure 2).

There was mean 6.5 (SD 1.0) days of digital wearable device monitoring during treatment with ranolazine (independent of randomization sequence) with 7.6% (16/210) days missing data and mean 6.6 (SD 0.9) days of activity monitoring during treatment with placebo (independent of randomization sequence) with 6.2% (13/210) days missing data. Pertinent baseline demographics and clinical variables are summarized in Table 1. Overall, the group mean was in the “low active” category of daily physical activity [31].

### Compliance and Safety

Compliance to the intervention, measured by returned pill counts, was available for 56 of 60 sessions and was 100% for those 56 sessions overall. Ranolazine and placebo interventions were well tolerated with three (ranolazine) and three (placebo) participants reduced to 500 mg twice daily dosing for adverse effects, as per protocol. No serious adverse events during the ranolazine period occurred. Nonserious adverse events during the ranolazine period occurred in two participants—nausea and lightheadedness (n=1) and excessive sweating (n=1)—and in one participant during placebo—throat swelling (n=1)—as previously reported [30]. No digital wearable device adverse events occurred; compliance to wearing the device and device function were verified at the end of the trial. One participant had very low step counts but was compliant with wearing the device and was kept in the analysis.

**Table 1:** Baseline demographics and clinical variables (N=30).

Variables		Analyzed participants	
Age in years, mean (SD)		54.03 (10.59)	
Female, n (%)		29 (97)	
Postmenopausal (n=29), n (%)		20 (69)	
**Body mass index in kg/m^2^ (n=29), mean (SD)**		27.71 (6.74)	
	>30 kg/m^2^, n (%)	10 (35)
Race (non-Caucasian), n (%)		25 (83)	
**Tobacco use, n (%)**			
	Current	1 (3)
	Former	8 (27)
	Never	21 (70)
Hypertension, n (%)		16 (53)	
Hyperlipidemia, n (%)		13 (43)	
Family history of premature coronary artery disease, n (%)		19 (63)	
**Coronary reactivity testing, mean (SD)**			
	Qualifying coronary flow reserve (n=13)	2.38 (0.63)
	Qualifying coronary blood flow (n=6)	90.82 (71.18)
	Qualifying acetylcholine response (n=10)	–0.08 (12.25)
	Qualifying nitroglycerin response (n=11)	19.31 (19.55)
	Qualifying cold pressor testing response (n=8)	7.94 (12.95)
	Left ventricular end-diastolic pressure (n=9)	13.33 (3.57)
**Cardiac magnetic resonance imaging, mean (SD)**			
	Baseline systolic blood pressure (n=28)	123.61 (21.03)
	Baseline diastolic blood pressure (n=28)	68.79 (12.38)
	Baseline heart rate (n=29)	70.59 (11.38)
	Baseline rate pressure product (n=28)	8804 (2284)
	Global myocardial perfusion reserve index (n=11)	1.69 (0.20)
	Subendocardial MPRI (n=11)	1.52 (0.14)
	Subepicardial MPRI (n=11)	1.75 (0.27)
**Symptoms, n (%)**			
	Typical angina	12 (40)
	Shortness of breath	24 (80)
	Palpitations	15 (50)
	Nausea	8 (27)
**Medications, n (%)**			
	Beta blockers	12 (40)
	Calcium channel blockers	7 (23)
	Angiotensin-converting enzyme inhibitors	2 (7)
	Angiotensin receptor blockers	6 (20)
	Nitrates	10 (33)
	Prior ranolazine	4 (13)
	Statins	13 (43)
	Aspirin	15 (50)
	Diuretic	3 (10)
	Hormone replacement therapy (n=19)	4 (21)
	Vitamin D	10 (33)

### Daily Physical Activity, Angina, Quality of Life, Hemodynamics, and Cardiac Magnetic Resonance Imaging Results

Step counts on individual days ranged from 7 to 19,879 during ranolazine and 25 to 18,110 during placebo. The individual mean across days within the treatment period ranged from 68 to 14,465 during ranolazine and 228 to 13,311 during placebo. During the ranolazine period, participants had significantly lower mean daily step counts compared to placebo (Table 2) with mean daily step count for ranolazine compared to placebo increased in 20% (6/30) of participants, decreased in 57% (17/30), and unchanged in 23% (7/30). The step count result was similar when the one participant with very low step counts was not included (mean change in step counts for the 29 remaining participants was mean –860, SD 1706, 95% CI –1509 to –211, *P*=.01). None of the secondary outcomes, other SAQ subscales, or angina or nitroglycerin use diary differed during ranolazine versus placebo (data not shown), although tPFR was higher on ranolazine (Table 2). Similar to the parent trial, due to a relatively high variance in the baseline-treatment comparison, we also directly compared treatment periods, rather than change from baseline, and observed no difference in within-subject change in SAQ-7 or SAQ subscales (physical limitation, angina stability, angina frequency, treatment satisfaction, QoL), DASI, SF-36 (energy/fatigue), SF-36 (emotional), diary-reported angina frequency or diary-reported nitroglycerin usage change, MPRI, PFR, and tPFR. Consistent with the parent trial, pharmacological stress heart rate and rate pressure product were lower during ranolazine periods versus placebo (Table 2).

**Table 2:** Mean daily step count, Seattle Angina Questionnaire (SAQ), hemodynamic, myocardial perfusion reserve, and diastolic filling treatment effect.

Variables^a^		Ranolazine		Placebo			Treatment change			
		Mean (SD)	N	Mean (SD)		N	Mean (95% CI)		N	*P*
Mean daily step count		5756.49 (3075.51)	30	6593.29 (3393.44)		30	–836.8 (–1464.74, –208.86)		30	.01
**SAQ**										
	Physical limitation	69.35 (26.10)	28	65.18 (21.44)		28	2.01 (–4.04, 8.06)		27	.50
	Angina stability	55.17 (27.04)	29	54.17 (26.33)		30	–6.03 (–22.63, 10.56)		29	.46
	Angina frequency	55.86 (27.32)	29	56.33 (23.56)		30	–4.14 (–13.76, 5.48)		29	.39
	Treatment satisfaction	66.31 (23.61)	29	66.81 (23.72)		30	–3.09 (–12.62, 6.44)		29	.51
	Quality of life	50.29 (21.76)	29	45.56 (19.54)		30	1.72 (–4.80, 8.25)		29	.59
	SAQ-7	58.19 (24.43)	29	55.86 (19.85)		30	–0.64 (–6.74, 5.46)		29	.83
**Pharmacological stress**^b^										
	Heart rate (bpm)	97.6 (14.1)	30	104.4 (16.34)		30	–6.8 (–9.8, –3.7)		30	<.001
	SBP (mmHg)	126.37 (24.55)	30	128.23 (20.42)		30	–1.87 (–8.68, 4.95)		30	.58
	DBP (mmHg)	58.83 (15.00)	30	60.93 (15.47)		30	–2.1 (–7.5, 3.3)		30	.43
	Stress RPP	12275.97 (2745.26)	30	13292.47 (2543.46)		30	–1016.5 (–1860.2, –172.8)		30	.02
	Global MPRI	2.09 (0.75)	30	2.16 (0.71)		30	–0.08 (–0.43, 0.28)		30	.67
	MPRI Subendocardial midventricular	1.9 (0.9)	30	1.9 (0.7)		30	–0.03 (–0.43, 0.36)		30	.86
**Diastolic filling**										
	PFR (mL/s)	322.8 (128.9)	30	321.35 (116.98)		30	1.5 (–27.6, 30.5)		30	.92
	tPFR (ms)	171.75 (43.31)	30	158.41 (39.85)		30	13.34 (0.91, 25.76)		30	.04

^a^DBP: diastolic blood pressure; MPRI: myocardial perfusion reserve index; PFR: peak filling rate; RPP: rate pressure product; SAQ: Seattle Angina Questionnaire; SBP: systolic blood pressure; tPFR: time to peak filling rate.

^b^Pharmacologic Stress=adenosine or regadenoson infusion.

**Figure 3:**
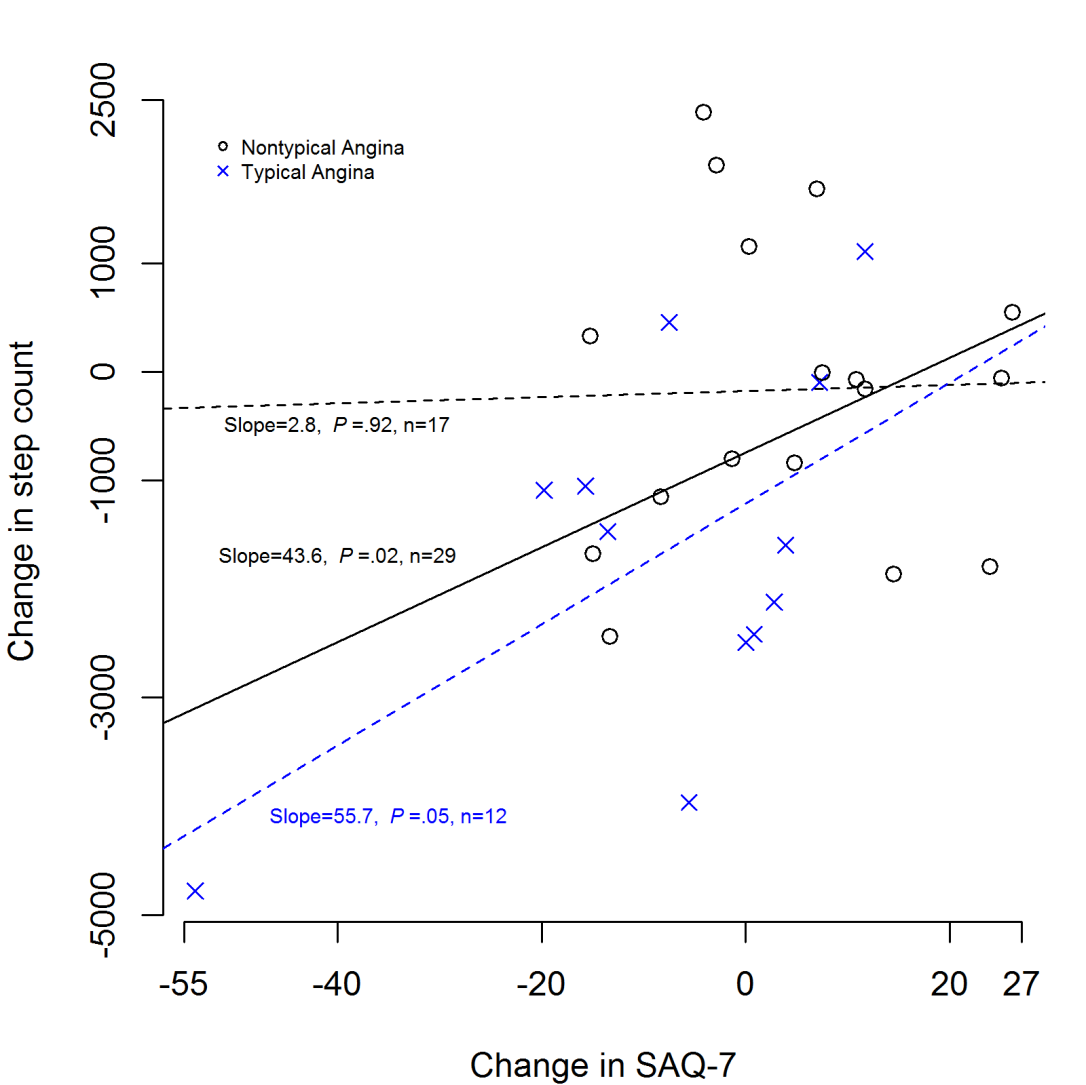
Correlation between change in angina and change in mean daily step count (n=29, *P*=.02). Patients who had higher mean step count on ranolazine are above the zero horizontal line and patients with a higher mean step count on placebo are below the zero horizontal line. Patients who had a larger treatment change under ranolazine are on the right of the vertical zero line and the participants who had a larger treatment change under placebo are to the left of the vertical zero line. The estimated regression line in this plot for all patients had an increasing slope (436 steps per 10 unit increase in SAQ-7 change, *P*=.02), also for the subgroup with typical angina (557 steps per 10 unit increase, *P*=.05).

There were no significant differences in baseline variables among patients who experienced a within-subject mean daily step count increase of 500 steps or greater (n=6), a decrease of 500 steps or greater (n=17), or a change of less than 500 steps (n=7). The change in 500 steps was informally based on the distribution of the step count data and a visual median *.* Increase in SAQ-7 (decrease in angina) from baseline (measured before initiation of treatment randomization and crossover), regardless of treatment, was directly correlated with a within-subject increase in mean daily step count (*r*=.42, *P=*.02) (Figure 2). This was more evident in participants with typical angina (*r*=.57, *P=*.05) compared to no correlation in participants with nontypical angina (*r*=.03, *P=*.92) (Figure 3).

There were no significant correlations for DASI, SF-36, diary-reported angina frequency and nitroglycerin usage, MPRI, and diastolic function with mean daily step count.

Subgroup analysis among the participants with typical angina (n=12) showed within-subject mean daily step count was lower during ranolazine compared to placebo (mean 5522 [SD 2891] vs mean 7150 [SD 3490], *P=*.007, respectively); however, there was no difference in step count among the participants with nontypical angina (n=18, mean 5913 [SD 3265] vs mean 6222 [SD 3376], *P=*.34, respectively). In participants with CFR less than 2.5 (n=9), mean daily step count trended lower during ranolazine treatment than placebo (mean 5298 [SD 1590] vs mean 6414 [SD 2862], *P=*.10, respectively). Further subgroup secondary measure analyses were not informative (data not shown).

Due to the relatively small sample size of the substudy and potential confounding variables, linear regression models used individual’s change in step count as the outcome between ranolazine and placebo and adjusted for various factors that may be associated with it, including age, body mass index, and typical angina versus nontypical angina. The model adjusting for age, body mass index, and typical angina versus nontypical angina did not identify any of these three factors to be significantly associated with change in step count.

When participants were categorized as “sedentary” daily physical activity [31] during the placebo treatment period of the crossover, observed mean daily step count was not significantly different by intervention during treatment with ranolazine compared to during treatment with placebo (mean 2752 [SD 1275] vs mean 2986 [SD 1384], *P*=.30, respectively); however, among participants whose daily physical activity level during the placebo treatment period of the crossover exceeded the category of “sedentary” daily physical activity [31], within-subject mean daily step count was lower during treatment with ranolazine compared to during treatment with placebo, but this difference was not statistically significant (mean 7259 [SD 2558] vs mean 8397 [SD 2529], *P=*.06, respectively).

## Discussion

We found in this substudy that late sodium channel inhibition was associated with a decreased step count overall during daily life in these participants, although the subgroup with angina improvement had a step count increase. This suggests that digital wearable device technology may be useful as a clinical variable and outcome in clinical trials research.

These substudy results are consistent with primary findings of the parent trial (eg, late sodium current blockade with ranolazine did not improve overall group angina in participants with CMD). Surprisingly, we observed a significantly lower mean daily step count during late sodium current blockade compared to placebo in our substudy. This finding is new and differs from the previous studies of ranolazine in CMD [28,29,17,30], although previously one clinical trial utilized a wearable accelerometer and demonstrated that treatment of heart failure with preserved ejection fraction with nitrates was associated with decreased daily activity [42]. Although our findings may offer potential insight for identification of patient subgroups who may benefit from antianginal therapies, these results must be considered exploratory given the small sample size of our substudy and do not modify existing clinical practice.

A variety of explanations may have contributed to the finding of reduced step count. The result could be a chance finding; however, it was present in the overall and subgroup analyses. It is possible that in CMD patients, those who are more active may have become less active due to unreported ranolazine side effects (ie, dizziness or gastrointestinal intolerance). In fact, two of the three reported side effects occurred during treatment with ranolazine. It is also possible that the digital wearable device was unable to detect the various types of daily activities participants may have performed, such as swimming or bicycling versus walking, or changes in daily activity intensity. Additionally, our results failed to detect a relationship between change in SAQ angina or QoL and change in step count, although our substudy was underpowered to evaluate this.

The unique attributes of our population and description of their daily activity should be noted. Women comprised 97% of our population, precluding conclusions regarding response in men, who typically are more represented in pivotal chronic angina trials with ranolazine (75%) and suffered from obstructive CAD [23,24,43]. To our knowledge, this report is the first time that levels of daily activity determined by a digital wearable device have been described in a CMD population.

### Strengths

The strengths of our substudy include the crossover design, which allowed participants to serve as his or her own control thus negating the effects of daily activity intersubject variability and reducing confounding effects of other antianginal therapies. Participant compliance with study medication is also a notable strength of our substudy.

### Limitations

The limitations of our substudy include a small sample size and a mean “low active” [31] cohort of participants. Although it is understandable that highly symptomatic angina patients limit their activity, this may make it more difficult to detect changes in activity. The SAQ has not been validated in CMD patients. Further, the 2-week active intervention duration may have minimized the measurable effect of late sodium channel inhibition (ranolazine) in the second week of monitoring, but this duration has been documented successfully in pivotal and dose-finding trials with ranolazine [23,44]. In addition, although participants verbally reported to research coordinators that they wore the device during the entire monitoring period as instructed per protocol, we did not monitor usage objectively; given randomization, we assumed that it was worn similarly in both groups. Finally, self-reported physical activity data was not collected to compare to data generated by the digital wearable device.

### Future Directions

Digital wearable devices have the potential for an expanded role in research as a clinical trial outcome and should be explored in a variety of health conditions.

### Conclusions

We report one of the first studies to use digital wearable device-determined step count as an outcome variable in a placebo-controlled crossover trial of late sodium channel inhibition in patients with CMD. Our substudy demonstrates that late sodium channel inhibition was associated with a decreased step count overall, although the subgroup with angina improvement had a step count increase. Our findings suggest digital wearable device technology may provide new insights in clinical trial research.

## Abbreviations

CAD: coronary artery disease
CFR: coronary flow reserve
CMD: coronary microvascular dysfunction
DASI: Duke Activity Status Index
ECG: electrocardiogram
MPRI: myocardial perfusion reserve index
MRI: magnetic resonance imaging
NHLBI: National Heart, Lung, and Blood Institute
PFR: peak filling rate
QoL: quality of life
SAQ: Seattle Angina Questionnaire
tPFR: time to peak filling rate
WISE: Women’s Ischemia Syndrome Evaluation
